# Somatic Embryogenesis in *Centaurium erythraea* Rafn—Current Status and Perspectives: A Review

**DOI:** 10.3390/plants10010070

**Published:** 2020-12-31

**Authors:** Ana D. Simonović, Milana M. Trifunović-Momčilov, Biljana K. Filipović, Marija P. Marković, Milica D. Bogdanović, Angelina R. Subotić

**Affiliations:** Department for Plant Physiology, Institute for Biological Research “Siniša Stanković”—National Institute of Republic of Serbia, University of Belgrade, Bulevar Despota Stefana 142, 11060 Belgrade, Serbia; ana.simonovic@ibiss.bg.ac.rs (A.D.S.); biljana.nikolic@ibiss.bg.ac.rs (B.K.F.); marija.nikolic@ibiss.bg.ac.rs (M.P.M.); milica.bogdanovic@ibiss.bg.ac.rs (M.D.B.); heroina@ibiss.bg.ac.rs (A.R.S.)

**Keywords:** antioxidative enzymes, arabinogalactan proteins, centaury, Gentianaceae, in vitro culture, morphogenesis, plant growth regulators, somatic embryo, tissue culture

## Abstract

*Centaurium erythraea* (centaury) is a traditionally used medicinal plant, with a spectrum of secondary metabolites with confirmed healing properties. Centaury is an emerging model in plant developmental biology due to its vigorous regenerative potential and great developmental plasticity when cultured in vitro. Hereby, we review nearly two decades of research on somatic embryogenesis (SE) in centaury. During SE, somatic cells are induced by suitable culture conditions to express their totipotency, acquire embryogenic characteristics, and eventually give rise to somatic embryos. When SE is initiated from centaury root explants, the process occurs spontaneously (on hormone-free medium), directly (without the callusing phase), and the somatic embryos are of unicellular origin. SE from leaf explants has to be induced by plant growth regulators and is indirect (preceded by callusing). Histological observations and culture conditions are compared in these two systems. The changes in antioxidative enzymes were followed during SE from the leaf explants. Special focus is given to the role of arabinogalactan proteins during SE, which were analyzed using a variety of approaches. The newest and preliminary results, including centaury transcriptome, novel potential SE markers, and novel types of arabinogalactan proteins, are discussed as perspectives of centaury research.

## 1. Somatic Embryogenesis: Biotechnological Exploitation of Plant Cells’ Totipotency 

Plants have unique developmental plasticity, which allows their adaptation to constant environmental changes. Plant in vitro culture techniques relies on this plasticity to mold the morphogenetic paths in the desired direction. Morphogenetic processes enabling the regeneration of the whole plant in in vitro tissue culture conditions are somatic embryogenesis (SE), organogenesis, micropropagation, androgenesis, and gynogenesis. Differentiated somatic cells grown in vitro begin to divide and can regenerate the whole plant through SE or organogenesis [1]. SE is the process during which somatic cells, under inductive conditions, form embryogenic cells that undergo morphological and biochemical changes leading to the formation of a somatic embryo [2]. SEis a powerful biotechnological method for the propagation and genetic improvement of many plant species, as it enables the obtaining of a large number of somatic embryos, which can be further used in the production of artificial seeds with diverse applications in biotechnology [3].

Somatic embryos can develop from a wide range of differentiated cell types, such as ovule, embryo, root, leaf, and meristem cells, in response to different exogenous and/or endogenous factors [4]. The presence and level of endogenous factors (phytohormones) determine whether SE can occur spontaneously, on a hormone-free medium, or must be induced by the addition of plant growth regulators (PGRs). SE can be direct (DSE), without an intermediate callusing phase, or indirect (ISE), which implies the formation of disorganized callus tissue [5]. Somatic embryos developed through DSE or ISE may be of uni- or multicellular origin [6]. 

Somatic cells are not totipotent per se, and they need induction under appropriate conditions [7]. During the induction phase, somatic cells acquire embryogenic competence and proliferate, while during the expression phase, embryogenic cells differentiate into somatic embryos [8]. These two phases are thought to be mutually independent and influenced by different factors. Competent cells represent a transition from somatic to embryogenic state, which still requires exogenous stimuli, while the embryogenic cells have the ability to regenerate embryos without exogenous stimuli [8,9]. Inductive conditions, such as exogenously added PGRs and stress factors, lead to the dedifferentiation of plant cells and activation of the embryogenic pathway [10]. It is still unclear why and how differentiated plant cells become totipotent and acquire embryogenic potential and why this phenomenon occurs only in certain plant species, certain tissue types, or cells [10]. Many genes are involved in the vegetative to embryogenic transition. These include phytohormone-responsive genes, such as auxin-related and ABA-inducible genes, genes involved in the cell cycle control, genes involved in growth and remodeling of the cell wall, as well as an array of transcription factors. The involvement of *LEC* (leafy cotyledon), *BBM* (baby boom), *WUS* (wuschel), *CLV* (clavata), *STM* (shoot meristemless), *SHR* (short root), *ABI3* (abscisic acid-insensitive), *FUS3* (fusca) and other transcription factorshave been confirmed in SE regulation [11]. Somatic Embryogenesis Receptor-Like Kinases (SERKs) are well-known SE-specific signaling components [12]. Although significant progress in identifying factors involved in induction, perception, and signal transduction during SE has been made, the results of these numerous studies are still fragmentary and insufficient to explain the events occurring during SE at the molecular level.

While the initiation of embryogenic tissues depends on the developmental stage of the used initial plant material and components of the nutrient medium, the sustention of the embryogenic potential during subsequent cultivation requires the simultaneous activity of signaling and genetic pathways. Identification of proteins and genes involved in the control of the embryogenic potential of the plant cells represents one of the most efficient ways for understanding the molecular mechanisms of SE. These molecules, so-called SE markers, can allow the identification of the cells with embryogenic potential in the tissue culture before visible morphological changes. Several proteins isolated during SE are stress-related or pathogen-related proteins. These proteins were isolated from different plants during stress treatments of plant tissue culture imposed by wounding, desiccation, heavy metals, or PGRs [13]. Extracellular proteins are also potentially good markers of SE because they play an important role in plant cell differentiation [14]. The largest number of extracellular proteins are glycoproteins, of which arabinogalactan proteins (AGPs) are especially important during SE. The current review covers several aspects of SE, including the influence of explant type and culture conditions (PGRs and light conditions), as well as the roles of antioxidative enzymes and AGPs, investigated in a medicinal plant *Centaurium erythraea.*

## 2. Centuries of Centaury Research 

*Centaurium erythraea* Rafn, commonly known as centaury, is a pharmacologically important medicinal plant from the Gentianaceae family. Centaury is a biennial, sometimes annual herb, which grows in wet to semi-arid areas throughout the northern hemisphere It is an ancient medicinal plant with the longest tradition in many pharmacopeias: it was described by Dioscorides nearly 2000 years ago. Centaury is used for the treatment of a wide range of ailments [15,16,17]. Using pure phytochemicals or crude plant extracts, experimental trials have been performed to evaluate anticancer, antioxidant, anti-inflammatory, antipyretic, analgesic, antimicrobial, antidiabetic, gastro-,cardio- and hepatoprotective activities of this important plant in many experimental animal systems [18,19,20,21,22,23,24]. A wide range of bioactive compounds can be found in the aerial part of *C. erythraea*, including secoiridoids, indole alkaloids, phenolic compounds (xanthones, flavonoids, and phenolic acid), and terpenes [25,26,27]. Centaur can also be used in the food processing industry as a natural flavoring or additive [26].

The ever-expanding demand for centaury in traditional medicine and the pharmaceutical industry has led to its uncontrolled collection resulting in a rapid decline of its natural populations. Development of methods for in vitro mass propagation of the centaury plants, as well as strategies for biotechnological production of its active metabolites, have attracted the attention of several research groups, resulting in a number of publications.

*C. erythraea* is relatively easily manipulated in the in vitro culture, where it can even complete its life cycle. Centaury’s manageability and developmental plasticity in vitro made it not only the most investigated species of the *Centaurium* genus but an emerging model in plant developmental biology. The diversity of morphogenetic paths that *C. erythraea* can undergo in vitro has been compiled recently [28]. The vigorous morphogenic potential of the explants favors the use of centaury for genetic transformations [29,30,31], interspecific hybridization in vitro [32,33,34], functional studies on secondary metabolite synthesis in vitro [25,26,35,36,37], and stress physiology studies [38,39]. Overall, different aspects of *C. erythraea’s* in vitro development, physiology, pharmacology, and ecology have been studied for over20 years at the Department for Plant Physiology, Institute for Biological Research “Siniša Stanković”, University of Belgrade, resulting in 38 journal papers, 8 book chapters, and 9 masters and doctoral theses. Similarities and differences between two in vitro systems for the induction of SE in centaury that have been extensively studied in our lab—spontaneous DSE from root culture and induced ISE from leaf explants—are the focus of this review. A newly developed system for secondary and cyclic SE is also described [40] and will be submitted as an accompanying article in this issue.

## 3. SE from Centaury Root Explants Is Spontaneous and Direct

Both SE and organogenesis in vitro can proceed either spontaneously, on hormone-free media, or be induced by (a combination of) PGRs. PGRs exogenously added to the nutrition medium, as well as the content of endogenous hormones in different plant tissues, affect the induction of SE [8,9,41]. Auxins and cytokinins (CKs) are the main factors that determine the response and direction of SE by controlling the cell cycle, activation of cell divisions, and cell differentiation [5,8], so their presence is required for SE induction [42,43,44]. However, only certain auxins, such as 2,4-dichlorophenoxyacetic acid (2,4-D) or naphthaleneacetic acid (NAA), were key factors for inducing embryogenic cells from immature leaves of *Manihot esculenta* [45]. In addition, 2,4-D or picloram (PIC) induced direct SE in leaf segments of *Petiveria alliacea* [46]. On the other hand, there are reports describing that CKs, such as thidiazuron (TDZ) and 6-benzylaminopurine (BA), played a crucial role in SE form leaf and shoot explants of *Ochna integerrima* and leaf explants of *Primulina tabacum* [47,48].

The first successful induction of SE in centaury was obtained in a cell suspension derived from callus cultures [49]. The calli were initiated from roots and shoots of seedlings on medium supplemented with kinetin (KIN, 10^−6^ M) and auxins indole-3-acetic acid (IAA, 10^−5^ M) or 2,4-D (10^−6^ M). In contrast to 2,4-D, IAA showed a stimulatory effect on SE induction from the cell suspension, but light was the main embryogenesis-inducing factor in this system.

**Figure 1:**
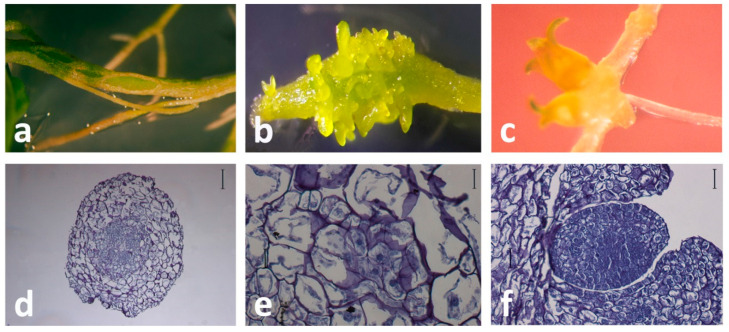
Direct somatic embryogenesis (SE) in *Centaurium erythraea* solid root culture. (**a**) The first response of root explant is enlargement and clear morphological changes observed five days after the culture setup on half-strength Murashige and Skoog (½MS) medium, (**b**) Detail of root explant with somatic embryos, (**c**) Cotyledonary somatic embryos developed directly from the root explant with no intervening callus phase, (**d**) Cross-section of root explant at the beginning of the culture. Scale bar indicates 200 μm, (**e**) Histological appearance of a proembryogenic structure. Scale bar indicates 100 μm, (**f**) Somatic embryo originated directly from the epidermal and subepidermal cells of the root tissue. Scale bar indicates 100 μm.

Subotić et al. [35] achieved the induction of SE from centaury root culture on solid half-strength Murashige and Skoog medium (½MS) without the addition of PGRs in the light. The somatic embryos developed alongside adventitious buds, so somatic embryos and adventitious buds of different developmental stages could be observed on the same root explant. Histological studies revealed that the somatic embryos formed directly from the epidermal cells, without the callusing phase, while adventitious buds developed from root cortex tissue [50]. In other words, SE from centaury root explants was spontaneous, direct (DSE), asynchronous, and occurred simultaneously with organogenesis. Somatic embryos derived from root explants of in vitro-grown centaury followed a unicellular pathway of DSE. These observations were in accordance with an earlier report describing in vitro morphogenesis from apical segments of primary hairy roots [29]. Even though the DSE from roots occurred spontaneously, the effects of PGRs added to the culture were further investigated. Subotić et al. [51] reported the effects of exogenous gibberellic acid (GA_3_) and paclobutrazol, an inhibitor of gibberellin synthesis, both added at concentrations 0.01–3.0 µM, on the SE induction in wild type and hairy root centaury culture. It was shown that GA_3_ had an inhibitory effect on the process of SE, while paclobutrazol in all applied concentrations had a stimulatory effect. The induction of SE in solid centaury root culture is presented in Figure 1.

As recently reviewed by Tomiczak et al. [52], SE has also been successfully obtained from the root cultures of several other species from the Gentianacea family, even though in these cases, the SE was not spontaneous as in centaury but required the addition of PGRs. Mikuła and Rybczyńsky [53] tried to induce SE in *G. cruciate* root explants cultured on MS medium supplemented with 2,4-D and kinetin. The root explants formed callus tissue at the cut surface precisely on the wounding site of roots. Further ultrastructural analysis has shown that the structures originating from single cortical cells resembled proembryos in root explants of *G. cruciate*, but the process of SE was not further continued [54]. On the other hand, in *G. kurroo*, *G. pannonica*, and *G. cruciate,* somatic embryos were regenerated by rhizodermal cells of adventitious roots [43]. This process was stimulated by various combinations of auxins and CKs, and somatic embryos were further converted into plantlets on a ½MS medium. Successful induction of SE was also achieved on root explants of *G. lutea* grown on a medium supplemented with auxins alone or in combination with cytokinin, although the conversion of somatic embryos into plantlets required the addition of mannitol or sorbitol to the basal culture medium [55]. The initiation of somatic embryos was also obtained in root explants of *Eustoma grandiflorum* cultured on a medium with 2,4-D [56]. The somatic embryos originated from pericycle and vascular parenchyma cells of seedling roots. Further conversion of the somatic embryos into plantlets was enabled with the addition of BA or GA_3_ [56].

## 4. Indirect SE from Centaury Leaf Explants

The successful induction of SE, as well as shoot and root regeneration in vitro, depends on a variety of factors, including the explant selection, light conditions, and exogenously added PGRs [5,57]. The leaf culture, implying in vitro cultivation of isolated leaves, is generally not used because the whole leaves cannot be maintained in tissue culture. However, if the leaf sections are used as initial explants, then calli, buds, or somatic embryos can be induced since some mesophyll cells have the potential to re-enter the cell cycle and become committed to different morphogenetic pathways when appropriately induced. Leaves from in vitro cultivated plants are an easily accessible source of explants, while the leaf culture enables the regeneration of genetically stable plants [58].

The effect of nutrient media and different PGRs on regeneration possibilities from centaury leaf explants have been investigated in several previous studies [59,60,61,62], but in all these reports, only adventitious buds and calli regenerated on the leaf explants. Recent research revealed that centaury leaf explants cultured on hormone-free medium in the light produced only a few shoots, while roots developed in darkness [28]. Both organogenesis and rhizogenesis occurred directly, without the callusing stage, but no somatic embryos developed on hormone-free media [28].

The first successful induction of SE from the centaury leaf explants was reported by Filipović et al. [28] on media containing *N*-(2-chloro-4-pyridyl)-*N’*-phenylurea (CPPU) and 2,4-D, applied together, where the embryogenic response increased with the increasing CPPU concentration. Synthetic urea-type cytokinin CPPU has a diverse morphogenic activity in different species [63]; other tested PGRs with cytokinin activity—6-benzyladenine, kinetin, and thidiazuron—induced callus proliferation only [28]. This combination of PGRs(CPPU + 2,4-D) induced somatic embryo formation in *Gentiana* spp. leaf explants, as well [43]. When the centaury leaf explants are cultivated on CPPU and 2,4-D, the direction of morphogenesis depends on the light conditions: If the explants are cultivated in darkness, the indirect formation of somatic embryos (ISE) is the only process that occurs, but when the explants are kept in the light, the processes of ISE and indirect shoot development (ISD) proceeded simultaneously, and both were asynchronous [28]. Even though ISE can be isolated from other morphogenetic paths by culturing the explants in darkness, a higher frequency of embryogenic callus induction was obtained in the light. Thus, it can be concluded that in centaury leaf culture, light is an obligatory factor for the organogenesis, but also a factor that enhances ISE [28], which is in accordance with previous reports where light-induced SE in centaury suspension culture [49], as well as the frequency of SE and the number of embryos per leaf explant of *Dendrobium* [64] and *Petiveria alliacea* cultures [46]. The developed somatic embryos originated from the leaf subepidermal cells [28].

**Figure 2:**
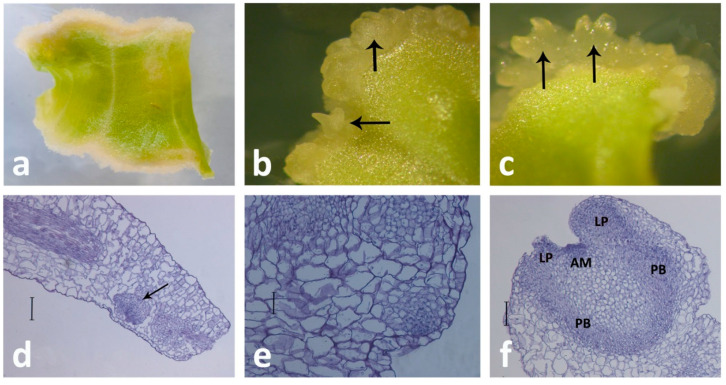
SE in *Centaurium erythraea* leaf culture. (**a**) Embryogenic callus developed at the edge of the leaf explant treated with 2,4-dichlorophenoxyacetic ac(2,4-D) and *N*-(2-chloro-4-pyridyl)-*N’*-phenylurea (CPPU) in darkness, (**b**) and (**c**) Somatic embryos at different stages of development (arrows), (**d**–**f**) Micrographs showing somatic embryo development on a leaf explant, (**d**) Histological appearance of a meristematic center (arrow) in the subepidermal layer of the leaf explant. Scale bar indicates 200 μm, (**e**) Globular somatic embryo. Scale bar indicates 100 μm, (**f**) Cotyledonary somatic embryo with apical meristem (AM), leaf primordial (LP), and provascular bundles (PB). Scale bar indicates 200 μm.

Plant regeneration via SE in leaf culture was also obtained in other gentian species. ISE in *G. pneumonanthe* was achieved on ½MS supplemented with 2,4-D and BA [65]. The embryogenic potential of leaf explants was also investigated in *G. kurroo*, *G. cruciata*, *G. tibetica*, *G. lutea*, *G. pannonica* [43]. The leaf explants of these species were grown on a medium supplemented with three auxins and five different CKs, and optimum regeneration was achieved in the presence of NAA in combination with BA or TDZ (thidiazuron). Furthermore, cytomorphological analyses have shown that somatic embryos originated from palisade mesophyll cells. SE was also induced on leaf explants of *G. straminea* and *G. utriculosa* cultured on an MS medium supplemented only with 2,4-D [66,67]. On the other hand, in leaf explants of *G. straminea*, *G. macrophylla,* and *S. chirata,* successful induction of embryogenic callus was achieved on medium with a combination of 2,4-D and CKs [68,69,70]. The process of ISE from the centaury leaf explants is illustrated in Figure 2 and the Supplementary video.

**Figure 3:**
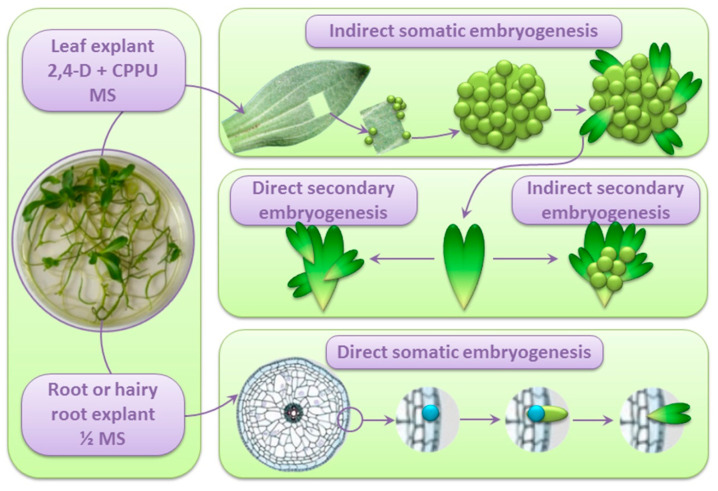
Schematic overview of SE in *Centaurium erythraea*. Left panel: Both leaves and roots of the in vitro grown *C. erythraea* plants can serve as sources of explants for the induction of SE. Hairy root cultures can also be used as explants. Upper panel: SE can be induced from leaf explants on the inductive medium containing 2,4-D and CPPU, both in the light and in darkness. Somatic embryos form from differentiated somatic cells in the subepidermal layer of the leaf explant. In this case, SE is indirect and proceeds via the callusing phase. Middle panel: The obtained somatic embryos can be further used as explants for secondary or cyclic embryogenesis [40] Lower panel: SE from root or hairy root explants is spontaneous, on ½MS medium and direct. SE starts with asymmetric divisions of single totipotent cells from the epidermal or subepidermal layers of root explant. Successive divisions give rise to somatic embryos.

Since the two main systems for the SE induction in centaury, DSE from root culture [35] and ISE from leaf culture [28], differ in their requirements regarding the addition of PGRs for the SE induction, the endogenous contents of different CKs, IAA, salicylic acid (SA) and abscisic acid (ABA) were analyzed in the roots and shoots of the in vitro grown plants as the sources of explants [71]. It was found that the total amount of endogenous CKs was 1.4 times higher in shoots as compared to shoots, but inactive or weakly active *N*-glucosides were the predominate CK forms in both organs, whereas free bases and O-glucosides represented only a small portion of the total CK pool. The roots were characterized with higher IAA content but lower IAA/free CK bases ratio and lower ABA content in comparison to roots. The most significant difference, however, was a 44-fold higher SA content in the roots as compared to shoots [71]. It is not clear which of these differences allows spontaneous SE from roots but not from shoots; for example, Quiroz-Figueroa et al. [2] demonstrated that very low concentrations of salicylates could induce cellular growth and enhance somatic embryogenesis in *Coffea arabica.* Planned investigation considering the determination of endogenous levels of phytohormones at different stages of somatic embryo development aims to relate these levels to the embryogenic capacity of centaury root and shoot explants. The processes of SE from centaury root and leaf cultures are presented in Figure 3.

## 5. Maintaining Reactive Oxygen Species Homeostasis during SE in Centaury: The Role of Antioxidative Enzymes

Three decades ago, Dudits et al. [72] suggested that the somatic embryo initiation in vitro was a stress response. Many reports since then underlined the importance of stress factors during SE induction in vitro [10,73,74,75,76]. Cultured plant tissues experience a variety of stresses as a consequence of in vitro manipulations, including wounding, sterilization, mineral nutrient imbalance in the culture medium composition, PGRs, or subcultures. In response to any of these stresses, the homeostasis between reactive oxygen species (ROS) production and scavenging is disturbed, and ROS are generated in excess, thereby imposing oxidative stress in plant tissue culture. Stresses experienced by cultured tissues may induce a general response, resulting in chromatin remodeling and activation of the embryogenic developmental program [72,74]. Namely, accumulating evidence revealed that ROS (specifically H_2_O_2_) may function as signaling molecules that regulate plant growth and development, including cellular proliferation and differentiation [77,78]. As a cellular messenger, H_2_O_2_ in proper concentrations has the ability to induce gene expression and protein synthesis, hence triggering activation of embryogenic developmental program and formation of somatic embryos in different plant species [76]. On the other hand, excessive ROS could severely damage cellular proteins, DNA, and membrane lipids [78]. Thus, ROS overproduction could lead to plant recalcitrance and reduced morphogenetic competence during the in vitro culture [79]. Therefore, maintaining an optimum ROS level in the cell and restoring cell redox balance is important and enables the regulation of various processes [78], including SE induction.

The level of H_2_O_2_ is controlled by the activities of several key enzymes, including superoxide dismutases (SODs), catalases (CATs), and class III peroxidases (POXs) [80]. The SODs provide the front-line defense against ROS since they scavenge superoxide radicals to produce H_2_O_2_ [81]. CATs remove the excess of H_2_O_2_, while extracellular POXs play a role in the precise regulation of ROS levels in the cell and apoplast because, in addition to their role in removing H_2_O_2_, they can also catalyze the formation of H_2_O_2_ and hydroxyl radicals [82,83,84]. By regulating ROS levels in the apoplast, POXs participate in cross-linking, cell wall reconstruction, and elongation [81]. In many plants, these antioxidant enzymes have been shown to play an important role in scavenging ROS that arise during SE [75,77].

To our best knowledge, the only study on the roles of antioxidative enzymes in relation to SE within the Gentianaceae family is the study on the already described system of regeneration and ISE induction from centaury leaf explants [28]. Filipović et al. [28] investigated the activities of SODs, CATs, and POXs in a comprehensive set of samples comprising intact leaves, wounded explants, and explants grown either in light or darkness on three types of media, of which one inductive medium (0.2 mg/L 2,4-D and 0.5 mg/L CPPU) supported ISE. Of these, only the changes in the antioxidative activities in response to wounding and during ISE will be discussed here.

Wounding of the centaury leaves (cutting the leaves into explants) caused an increase in SOD activity (comprising 3 Cu/Zn-SOD isoforms), an increase in CAT activity (comprising 3 major activity bands), as well as a decrease in total POX activity [28], indicating that SOD and CAT are involved in the protection of centaury leaves from wounding-induced oxidative damage. Wounding leads to an accumulation of ROS in *Medicago truncatula* leaf explants, which occurs within seconds [85]. Slesak et al. [86] showed that mechanical injury of *Mesembryanthemum cristallinum* leaves leads to H_2_O_2_ accumulation, which was accompanied by an increase in total SOD activity and a decrease in CAT activity. Decreased POX activity in response to wounding, recorded in centaury leaves, is consistent with low POX activity in freshly isolated leaf explants of *Dactylis glomerata* [1]. Mechanical wounding is an inevitable consequence of in vitro manipulations. Wound signaling triggers not only defense responses, such as the production of ROS but also healing responses, including dedifferentiation, cell cycle reactivation, and vascular regeneration [87].

Following rapid responses to wounding, ROS homeostasis has to be reestablished, which is crucial for initial cell dedifferentiation and division during callus formation [88]. Subsequent planting of the centaury leaf explants on inductive medium strongly induced total POX activity, both in light and darkness, suggesting the importance of these enzymes in cell division, growth, and differentiation, probably through their action on cell wall remodeling [82]. A statistically significant increase in POX activity in comparison to the control intact leaves occurred after seven days of incubation, when the first cell divisions and the formation of meristem centers in the sections of the centaury leaves were observed, with the peaks of POX activity on the 14th or 21st day in culture, which coincidence with the emergence of somatic embryos. Therefore, it could be concluded that POXs play an important role in the development of centaury somatic embryos. Previous reports confirmed the important role of POX during SE induction from leaf explants of *D.* *glomerata* [1] and *Cicer arietinum* [44]. On the contrary, SOD activity decreased in light and remained unchanged during ISE in darkness, while CAT activity decreased during ISE both in light and darkness. The obtained results illustrate that dynamic changes in the antioxidative enzymatic capacity upon wounding and in response to SE induction are required to maintain ROS homeostasis in centaury leaf explants.

## 6. Studies on the Role of AGPs during SE in Centaury Using β-D-glucosyl Yariv Reagent

AGPs are heavily glycosylated, intrinsically disordered glycoproteins ubiquitous in plants, which belong to a superfamily of cell surface hydroxyproline-rich glycoproteins (HRGPs) [89,90]. The extraordinary structural diversity of AGPs relies not only on their protein backbones encoded by large gene families [89,91] but also on the possibility of differential glycosylation of the same isoform into hetero generous glycoforms [92]. Structural features that are common to AGPs include the presence of branched type II arabino-3,6-galactans (AGs) and short oligoarabinosides(both *O*-linked to the hydroxyproline (Hyp) residues), a high percentage of amino acids that constitute the AG-II glycomodules (Pro/Hyp, Ala, Ser, Thr, and Gly), N-terminal signal peptide directing their synthesis via secretory pathway, and often a C-terminal glycosylphosphatidylinositol (GPI) lipid anchor signal peptide [93,94]. While many AGPs are GPI-anchored to the plasma membranes, others may be secreted to the medium [93,95]. Dragićević et al. [89] recently demonstrated that many AGP sequences may have transmembrane domains. Beside these basic structural features’ characteristic for classical AGPs and their short counterparts, AG peptides, many AGPs contain additional conserved domains or functional motifs and are termed chimeric AGPs [94].

AGPs are involved in cell proliferation [96] and diverse developmental and physiological processes, including differentiation and patterning [93,95,97]. Involvement of AGPs in SE has been described in many plant species, such as maize [98], chicory [99], *Trifolium nigrescens* [100], and others. As discussed below, the role of AGPs during SE from centaury roots [101,102] and leaf explants [103,104] has been investigated using a variety of approaches. One of the main tools for studying the AGPs’ functions, used for decades, is a synthetic red dye, β-D-glucosyl Yariv reagent or βGlcY [105]. Most AGPs specifically bind βGlcY [1,3,5-tris (4-β-D-glycopyranosyloxyphenylazo)-2,4,6-trihydroxybenzene]; for β-galactosyl Yariv reagent, similar to βGlcY, a noncovalent interaction with β-1,3-galactan moieties of AGPs has been demonstrated [106].

**Figure 4:**
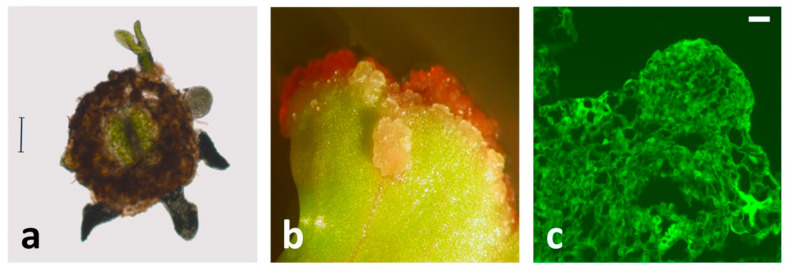
Distribution of AGPs during SE in centaury. (**a**) Cross-section of a root explant with somatic embryos at its surface, stained with βGlcY reagent. Scale bar indicates 80 μm (**b**) Indirect SE on centaury leaf explants grown on 100 μM βGlcY reagent in darkness. Somatic embryos form only on the parts of the explants that are not in direct contact with the medium. (**c**) Embryogenic globule developed on leaf explant and labeled with JIM15 antibody. Scale bar indicates 10 μm.

βGlcY has been widely used as a histochemical reagent to detect AGPs int issue sections [107]. When *C. erythraea* roots are used for SE induction on a hormone-free medium, initially, the whole root explants were stained with βGlcY, but the most intense staining was in the epidermal cells and vascular tissue [101,102]. After one week in culture, βGlcY intensively stained AGPs in the surface cell layers of the centaury root explants, where somatic embryos were likely to develop. A similar staining pattern was observed in the outer epidermal cells during SE induction in chicory root culture [99]. Considering that somatic embryos originate directly from the root epidermal cells [50], the accumulation of AGPs in this region is indicative of their involvement in SE initiation. After two weeks in culture, the subepidermal layers of root explants also reacted with βGlcY, but neither developing globular embryos nor adventitious buds (which form alongside the embryos) showed significant precipitation of AGPs with βGlcY [102]. Finally, after 8 weeks in root culture, the epidermal and subepidermal cells were deeply stained with βGlcY, while staining of vascular tissue was less intense. This is shown in the root cross-section (Figure 4a), where several developed somatic embryos, as well as adventitious buds, can be seen.

To study the role of AGPs during morphogenesis in vitro, βGlcY can be applied as an adjuvant to the culture medium [96,99,108,109]. Inactivation of AGPs by βGlcY binding during the induction of SE in different systems, commonly inhibits SE [99] and/or affects embryos’ development and morphology [108,109]. As expected, the addition of βGlcY to the inductive medium during the induction of ISE from centaury leaf explants in darkness reduced the number of developed somatic embryos per explant in a dose-dependent manner [103]. The concentration of 150 µM βGlcY almost completely inhibited ISE, whereas, at lower concentrations, the embryos developed only in the explants’ regions that were not in direct contact with the βGlcY-containing medium (Figure 4b). Indirect shoot development on the same inductive medium in the light or direct shoot development on a hormone-free medium were also inhibited by βGlcY in a concentration-dependent manner but were less sensitive to βGlcY than ISE [103]. The obtained results clearly point at AGPs as essential factors during ISE from centaury leaf explants.

However, quite unexpected results were obtained when βGlcY was used to investigate the role of AGPs during morphogenesis (simultaneous development of somatic embryos and adventitious buds) from centaury root explants [101,102]. Namely, it turned out that βGlcY may actually stimulate the morphogenesis from the root explants, albeit not in a linear dose-response manner. βGlcY increased the shoot regeneration frequency of roots cultured on the hormone-free medium from 71.67% for untreated culture to 93.89% and 92.22% for cultures grown on 15 µM or 25 µM βGlcY, respectively. The same concentrations also increased the average number of regenerated shoots per root explant, while lower (5 µM) or higher (50–75 µM) βGlcY concentrations had little effect on the regeneration potential of the 8-week-old root culture in comparison to untreated control [101]. Comparable results were obtained when the regeneration was scored after four weeks in culture [102] or when 1 µM IBA was added to the medium [100]. In any case, the obtained regenerants displayed normal morphology. Interestingly, the shoots regenerated on media containing 25–75 µM βGlcY had elevated content of AGPs in comparison to control shoots [101], as determined by the single radial gel diffusion method, which also utilizes βGlcY [110]. The roots developed on regenerated shoots also had increased AGP levels when developed on βGlcY-containing media [101]. This finding suggests that blocking of AGPs may increase their synthesis by some type of feedback regulation. The authors suggested that βGlcY in tissue culture may act as a stressor that may stimulate regeneration [102] since βGlcY triggers wound-like responses in *Arabidopsis* cell culture, as shown by whole-genome array [111]. On the other hand, the presence of 75 µM βGlcY in the centaury 4-weeks old leaf culture did not alter the AGP content, regardless of other conditions (basal or inductive medium and light vs. darkness) [103], so the effect of βGlcY on AGPs accumulation might be tissue-specific. Finally, the profile of AGPs present in the regenerating leaf explants, as determined by crossed electrophoresis [112], depends on the medium composition, light conditions, and culture age [103].

## 7. Dynamic Changes of AGPs Distribution and Expression during SE in Centaury

Overall, the evidence collected using βGlcY reagent in different assays suggests that AGPs are important for the induction of SE in centaury. However, tracking the dynamicsof AGPs’ distribution during regeneration requires more sophisticated methods, such asa widely use dimmunohistochemical approach with monoclonal antibodies (mAbs) raised against AGPs’ carbohydrate epitopes.A large set of the anti-AGP mAbs of the JIM, LM, and MAC series are commercially available and are listed, along with the epitopes they recognize, in several reviews [95,96]. The exact structure of the recognized epitopes is not always clearly determined, but it is known that MAC207, JIM4, and JIM13 bind to the β-d-GlcA-(1→3)-α-d-GalA-(1→2)-α-l-Rha motif, whereasLM2 recognizes β-linked glucuronic acid (β-D-GlcA). A systematic review of the available literature describing the expression of different mAb-recognizable AGP epitopes during SE in different species [98,99,100,109], to mention a few, would surely transcend the scope of the current review and would only confirm that none of the tested epitopes stands out as a universal SE marker in all or most of the studied plant species. Thus, the existence of two systems for SE induction in the same species—DSE on the hormone-free medium from root explants [102] and ISE from the leaf explants cultured on inductive medium [104]—provides an opportunity for the comparison of the obtained immunohistochemical results and search for common epitope markers or patterns.

The morphogenesis from the root explants was studied using LM2, JIM13, JIM15, JIM16, and MAC207 mAbs, of which the expression of JIM13-reactive epitope was not detected at all [102]. The JIM16 epitopes were localized in all cells of the root explants, especially in the endodermis and the central cylinder, as well as in the newly formed meristematic centers, so they were considered as markers of organogenesis, not somatic embryogenesis. The remaining three mAbs reacted with the epitopes present in somatic embryos. LM2 epitopes were widely distributed in root cross-sections at the beginning of the culture, but after 4 weeks in culture, the LM2 signal was more localized in epidermal cells and newly formed globular somatic embryos [102]. Comparable results for LM2 localization were obtained during DSE from chicory roots [99], where this epitope was foundin the surface cell layer surrounding somatic embryos; however, in this system, JIM13 and JIM16 mAbs were also expressed. MAC207 epitope had strong expression in protodermal cells of the embryos, as well as at the surface of epidermal cells of root explants adjacent to globular somatic embryos, with a strong signal in the extracellular matrix. Finally, the JIM15 epitope was reactive with AGPs in developed somatic embryos, as well as in the cells of the vascular elements of the root explants [102].

A slightly different set of mAbs, comprisingJIM4, JIM8, JIM13, JIM15, LM2, LM14, and MAC207, was used to investigate the distribution of the corresponding AGP epitopes during ISE from *C. erythraea* leaf explants [104]. As discussed above, in this system, light induces simultaneous development of both somatic embryos and adventitious buds, whereas in darkness, only ISE occurs. Generally, in globular somatic embryos, a different distribution pattern of JIM4, JIM13, JIM15, LM2, and MAC207 epitopes was observed, while with the progression of SE, the number of detected AGPs decreased. When the explants are cultivated in darkness, the JIM4 epitope was strongly expressed from the earliest stages of SE: It localized in the epidermal and subepidermal cells which formed meristematic centers, in the embryogenic cells in meristematic calli, as well as in four-cell proembryo. During further proembryo development, strong expression was detected only in the extracellular matrix surrounding the proembryogenic nodule. At the globular stage, JIM4 epitopes were found in the cell walls of the protodermal cells, while at the early cotyledonary stage, the JIM4 fluorescence was moderate [104]. Even though this mAb was not exclusively present in embryogenic tissues, its expression in adventitious buds formed in the light was weak. In maize callus culture, JIM4 was as also an early marker of embryogenic competence [98]. A similar labeling pattern to JIM4 in globular somatic embryos was found for MAC207 since its strong signal was detected in the cell walls of protodermal cells [104], just as was seen in protodermal cells of the globular embryos developed from roots [102].The JIM13 epitope, which was not observed during SE from the root explants at all [102], showed an intense signal in the whole globular embryos developed from leaf explants in darkness but was not restricted only to the embryogenic tissues [104]. The expression of JIM13 decreased in late embryos. In adventitious buds developed in the light, this epitope was not detected. Strong JIM13 labeling was also found in the embryogenic sector during SE in peach palm, where it was associated with extracellular matrix surface network [108]. Likewise, high-intensity JIM15 (Figure 4c) and LM2 fluorescence were localized to the whole globular embryos, but not in later developmental stages. A strong LM2 signal was observed in the cell walls of meristematic cells from which somatic embryos develop and in cells of embryogenic swellings in *Trifolium nigrescens* [100]. Both JIM15 and LM2 signals were also seen in developing adventitious buds, so they were not an exclusive feature of SE. Unlike other tested epitopes, which appeared early during ISE, the LM14 signal was not present in globular somatic embryos but was strong and evenly distributed throughout the longitudinal sections of the heart embryo. Finally, theJIM8 epitope was detected in the extracellular matrix, as well as in adventitious buds, but not in somatic embryos of any stage [104].

The obtained immunohistochemical results only corroborated well-established observations that spatiotemporal occurrence of AGPs during SE is developmentally regulated and that AGPs may serve as positional markers, markers of cell identity, or markers for embryogenic competence [97,98,99,100]. Our results indicate that the profile of AGP epitopes expressed during SE is not only species-specific but also strongly depends on the explant type and the culture conditions: While some epitopes, such as MAC207, have similar expression patterns in both regeneration systems, others, such asJIM13, are strongly expressed in somatic embryos developed from leaves, but are absent in embryos regenerated on *C. erythraea* roots. Furthermore, even though some mAbs recognize the same epitope (MAC207, JIM4, and JIM13), they display different labeling patterns.

Even though anti-AGP mAbs have been widely used for studying AGPs’ distribution during SE and other developmental processes, their usefulness is intrinsically limited for several reasons: (1) mAbs are not specific for a single AGP; (2) they cannot distinguish all glycoforms of an AGP backbone, and (3) the epitope has to be unmasked for immunodetection [89,91,92]. Of course, that the analysis of the spatiotemporal pattern of gene expression can indicate the involvement of a particular AGP in some process [93], but in non-model species, such as *C. erythraea*, the necessary sequence resources are commonly unavailable. Thus, we initially used in-house assembled centaury leaf and root transcriptomes [113] and mined centaury AGP sequences using a homology-based search. Using this approach, we have identified four centaury AGP transcripts, named *CeAGP1* through *CeAGP4* [103]. Of these, *CeAGP1*, *CeAGP2,* and *CeAGP4* (GenBank: KC733882, KC733883, and KC733885, respectively) were characterized with conserved fasciclin domains and represented members of a subclass of chimeric AGPs known as fasciclin-like AGPs or FLAs [114]. *CeAGP1* was highly induced (26.7-fold) during morphogenesis from centaury leaf explants in the light, where ISE was accompanied with indirect shoot development, but more importantly, it was over 20 fold induced during ISE in darkness, in comparison to the control explants, indicating its importance during ISE [103]. *CeAGP2* was slightly induced during both direct (on hormone-free medium) and indirect morphogenetic paths (ISE and indirect shoot development on inductive media), while the induction of *CeAGP4* during ISE and organogenesis on inductive media was very low [103]. The role of *CeAGP1* in ISE can be viewed in light of the general role of FLAs as molecules involved in cell adhesion and protein–protein interactions [114]. *CeAGP3* is an AG peptide with a conserved DUF1070 domain (GenBank: KC733884, protein:AGN92423). The expression pattern of *CeAGP3* indicated its general involvement indifferent morphogenetic paths in centaury, since this transcript was induced 36.6-fold relative to control during ISE in darkness, but was also highly induced during indirect morphogenesis in the light (ISE and organogenesis), as well as in direct organogenesis on a hormone-free medium [103]. We have analyzed all 271 sequences containing the DUF1070 domain (DUF stands for Domain of Unknown Function) from 25 diverse families of vascular plants, aiming to elucidate the function of this motif. As it turned out, most of the DUF1070 domain represented typical glycosylphosphatidylinositol lipid anchor signal peptide (GPIsp) found in short AGPs (AG peptides), so the DUF1070 was renamed to arabinogalactan peptide (PF06376) [115]. To our best knowledge, DUF1070/PF06376 is the only conserved domain exclusively found in AGPs and HRGPs, in general. GPI anchors in proteins, such as *CeAGP3,* are proposed to increase lateral mobility of the anchored proteins in the plasma membrane, allow polarized targeting to the cell surface, inclusion in lipid rafts, as well as further processing by GPI-specific phospholipases and glycosidases, thereby releasing diffusible AGPs and/or carbohydrates as extracellular signals, as well as biologically active lipids as intracellular signals [91,94,95,97,103,115]; any of the proposed features for GPI-anchored AGPs may be important for morphogenesis and SE.

## 8. Perspectives: Novel SE Markers, “AGP-Tyr Kinases”, and Time-Laps Embryogenesis

To support the analysis of the molecular events during SE and other in vitro morphogenetic processes in centaury, we have recently sequenced six *C. erythraea* transcriptomes (embryogenic calli, globular somatic embryos, cotyledonary somatic embryos, adventitious buds, leaves and roots of in vitro grown plants) and de novo assembled referent transcriptome comprising 105.726 genes [116]. The high quality and completeness transcriptome were functionally annotated and made publicly available. The transcriptome, along with a set of validated housekeeping genes, comprises a framework for the search for genes involved in SE and organogenesis [116]. A subset of genes potentially involved is SE was selected as transcripts with ≥8-fold higher expression (FPKM values) in embryogenic tissues as compared to non-embryogenic tissues, and their expression was further analyzed by qRT-PCR in 16 tissue samples [117]. The most intriguing finding of this preliminary research was the expression profile an unknown gene (provisionally termed *UN1*), a 725 bp long transcript with no BLAST hits or homology with any known sequence. *UN1* was highly expressed in leaf-derived embryogenic calli, while its expression progressively decreased in globular and cotyledonary embryos. The *UN1* expression in seedlings, roots, leaf-derived adventitious buds and leaves from flowering plants was below the qPCR detection limits, implying that its expression is restricted to the initial ISE stages [11]. The investigation of *UN1* structure and function is ongoing; for now, we can only speculate that *UN1* may have an impact on the acquisition of the embryogenic potential, and as such it may be a novel SE marker.

**Figure 5:**
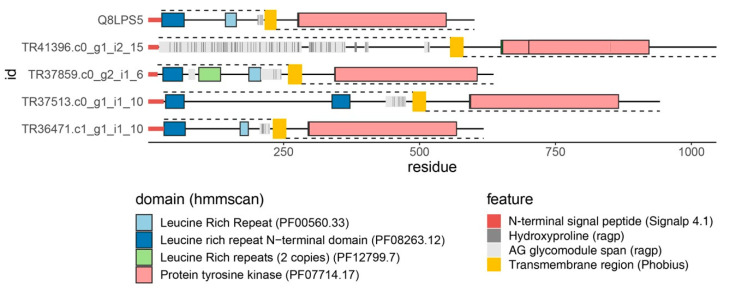
Arabinogalactan proteins (AGPs) with Tyr kinase domains. The first sequence is Somatic Embryogenesis Receptor-like Kinase 5 (SERK5) from *A. thaliana* (AT2G13800.1 or Q8LPS5 protein precursor). Four sequences below are found in the *C. erythraea* transcriptome, based on homology with SERK5. In addition to AG glycomodules with predicted hydroxyprolines and Tyr kinase domains, all sequences have N-terminal signal peptide and a transmembrane domain, while most have Leucine-rich repeats typical for SERK receptors. The image is generated using ragp.

As discussed above, a homology-based search revealed only four AGP sequences in the first version of the *C. erythraea* transcriptome, all of which were AGPs with conserved domains [103]. This was expected since HRGPs, including AGPs, are intrinsically disordered proteins lacking hydrophobic core, so the sequence constraints imposed on these proteins are relatively low. Therefore, AGPs can rapidly mutate and evolve, which hinders their homology-based mining [90]. We have recently developed a highly sophisticated bioinformatics pipeline developed in R, ragp, for mining and analysis of HRGPs with an emphasis on AGPs [90]. The key novelty incorporated in ragp is the machine learning-based prediction of proline hydroxylation sites, which represent the glycosylation sites. The analysis of *C. erythraea* transcriptome [118] as well as 62 plant proteomes using ragp [90] revealed, quite unexpectedly, that the most frequently identified domains found in AGPs were the Protein kinase and Protein tyrosine kinase domains. The Protein (tyrosine) kinase domains have thus far eluded experimental evidence for linkage with AGPs in any plant species. Possible implications of this finding include a novel way of attachment of AGPs to the plasm membrane through their transmembrane domains and a novel way for the involvement of AGPs in signaling. So far, structural features of AGPs and circumstantial evidence suggested that AGPs may be involved in signaling as co-receptors [97], or through interaction with membrane receptors (including protein kinases) on the same or neighboring cell [11,91,93], interaction with other AGPs, pectins, and other cell wall or cytoskeletal elements [89,93]. The presence of protein kinase domains on ragp-predicted AGPs suggests that AGPs may actually be the membrane receptors themselves or that certain Protein kinases have previously undetected AG-glycomodules and can be glycosylated. While the experimental evidence for the Hyp-glycosylation of these protein kinases is lacking, and the functions of the proposed “AGP-Protein kinase” molecules are unknown, it should be noted that, for example, *A. thaliana* SERK5 (AT2G13800.1) has predicted hydroxyprolines organized in characteristic AG-glycomodules [90,118] (Figure 5). The analysis of expression and function of “AGP-Protein kinases” and their possible involvement in SE in *C. erythraea* is planned.

Since both DSE from centaury roots [35,50] and ISE from leaf explants [28] are asynchronous, collecting embryogenic tissues, specifically somatic embryos at different developmental stages for molecular and biochemical analyses, is very tedious and time-consuming. Unfortunately, the establishment of a synchronized embryogenic culture has not been achieved in *C. erythraea* yet, and it remains one of our goals. A synchronized culture would not only aid the harvest of somatic embryos at a specific stage but would also indicate the exact timing to a specific developmental event under certain conditions. An alternative way to achieve this is documentation of the development of embryogenic structures on selected explants over time. Such documentation system has been established by the combination of photography (using a smartphone camera with a macro lens), image processing of focalstacks from the developing explants automated in Adobe Photoshop and Bridge, and a relational database built using Excel and R [119]. An example of such a time-lapse documentation video of SE from leaf explants is provided as a Supplement.

## 9. Conclusions

Even after 20 years of research, *Centaurium erythraea* remains an attractive, challenging, and yet rewarding experimental object at our Department. *C. erythraea* is already firmly established as a valued model system at our Department for the studies on alternative ways for the production of secondary metabolites and for the studies on morphogenesis in vitro—both primarily aimed at its conservation and sustainable usage. However, the accumulated data and successful protocols for the centaury propagation in vitro [28] have led us to gradually shift our focus from centaury’s potential as a medicinal plant to its possibly even greater potential as a genetic resource for crop improvement. Namely, we believe that centaury’s immense regeneration potential and developmental plasticity, when cultivated in vitro, rely on the presence or high activity of certain genes that may not be present or active in plant species recalcitrant to SE induction or in vitro propagation and manipulations in general. Having a sequenced *C. erythraea* transcriptome [116] would allow us, and other research groups interested in centaury development, to mine for genes that are highly active during SE and organogenesis, and hopefully find genes, such as *UN1* [117], that were not described before. Such novel genes, as well as known genes previously unassociated with morphogenesis, could be considered as sequence resources for the genetic improvement of valuable crops that are recalcitrant to in vitro manipulations. In addition, finding genes that are differentially expressed during DSE and ISE from roots and leaves, respectively, specifically AGP genes and genes associated with auxin and cytokinin signaling or metabolism, as well as differences in the endogenous hormones during these two processes, would probably highlight some factors governing SE via direct or indirect pathway. Thus, unrevealing at least a part of the molecular networks and genes that are at the base of SE induction and other regeneration processes in centaury is the primary focus for our future research.

## Supplementary Materials

The following are available online at https://www.mdpi.com/2223-7747/10/1/70/s1, Time laps photo-documentation of somatic embryogenesis from *Centaurium erythraea* leaf culture is provided in a form of a video file Centaury somatic embryogenesis.mp4. The size of the video is about 25 Mb.
